# Lexical Speech Features of Spontaneous Speech in Older Persons With and Without Cognitive Impairment: Reliability Analysis

**DOI:** 10.2196/46483

**Published:** 2023-10-10

**Authors:** Phillip Hamrick, Victoria Sanborn, Rachel Ostrand, John Gunstad

**Affiliations:** 1 Department of Psychological Sciences, Kent State University, Kent, OH, United States; 2 Rhode Island Hospital, Providence, RI, United States; 3 IBM Research, Yorktown Heights, NY, United States

**Keywords:** Alzheimer’s disease, cognitive dysfunction, early diagnosis, psychometrics, speech, technology assessment

## Abstract

**Background:**

Speech analysis data are promising digital biomarkers for the early detection of Alzheimer disease. However, despite its importance, very few studies in this area have examined whether older adults produce spontaneous speech with characteristics that are sufficiently consistent to be used as proxy markers of cognitive status.

**Objective:**

This preliminary study seeks to investigate consistency across lexical characteristics of speech in older adults with and without cognitive impairment.

**Methods:**

A total of 39 older adults from a larger, ongoing study (age: mean 81.1, SD 5.9 years) were included. Participants completed neuropsychological testing and both picture description tasks and expository tasks to elicit speech. Participants with T-scores of ≤40 on ≥2 cognitive tests were categorized as having mild cognitive impairment (MCI). Speech features were computed automatically by using Python and the Natural Language Toolkit.

**Results:**

Reliability indices based on mean correlations for picture description tasks and expository tasks were similar in persons with and without MCI (with *r* ranging from 0.49 to 0.65 within tasks). Intraindividual variability was generally preserved across lexical speech features. Speech rate and filler rate were the most consistent indices for the cognitively intact group, and speech rate was the most consistent for the MCI group.

**Conclusions:**

Our findings suggest that automatically calculated lexical properties of speech are consistent in older adults with varying levels of cognitive impairment. These findings encourage further investigation of the utility of speech analysis and other digital biomarkers for monitoring cognitive status over time.

## Introduction

### Use of Digital Biomarkers as a Method for Cognitive Monitoring

Much like monitoring cardiac rhythm through smartwatches, the integration of smart technology into the daily lives of older adults creates new opportunities for the remote monitoring of cognitive function. Researchers have started to use *digital biomarkers*, which are defined as “objective, quantifiable, physiological, and behavioral data that are collected and measured by means of digital devices, such as embedded environmental sensors, portables, wearables, implantables, or digestibles,” to help identify and track symptoms in persons with dementia [1].

### Speech Analysis Data as Digital Biomarkers

A growing number of digital biomarkers have been examined in persons with Alzheimer disease and related dementias (ADRD), such as home-based motion sensors and systems that monitor driving performance. Spontaneous speech appears particularly promising, presumably because the declarative memory system that supports some aspects of language [2] changes dramatically in persons with ADRD. Technological advances now allow commonly observed language changes in persons with ADRD (eg, wording-finding problems and empty speech) to be automatically computed from transcripts of spontaneous speech, and the resulting indices appear sensitive to early cognitive dysfunction. For example, lexical frequency, which quantifies an individual’s ability to access more versus fewer common words, has been shown to predict current and future cognitive status [3,4]. Other studies suggest that indices from spontaneous speech may be even more sensitive to ADRD than traditional neuropsychological language tests of confrontation naming or semantic fluency [5].

### Study Aims

Though such findings are encouraging, many practical questions remain regarding the feasibility of using spontaneous speech analysis to monitor cognitive function. A key concern is the limited investigation of the psychometric properties of speech features. Put simply, whether an individual’s spontaneous speech is internally consistent enough to be used as a marker of cognitive function has yet to be determined. Many person- and environment-based factors are known to influence spontaneous speech production (including age, sex, task demands, nativeness, and proficiency, among others [6,7]), and the degree to which a short sample of spontaneous speech reflects an individual’s general speech has not been previously examined. This study aims to provide a preliminary examination of the reliability of lexical features calculated from the spontaneous speech produced by older adults. That is, we were interested in determining how much variability or consistency was exhibited within and across these features. In effect, our analysis is analogous to examining the test-retest reliability of a traditional neuropsychological test. We hypothesized that speech features would be consistent both between multiple instances of a similar speech elicitation task and across different types of speech elicitation tasks in persons with and without mild cognitive impairment (MCI). In combination, these analyses provide critical insight into the appropriateness of using spontaneous speech indices to predict cognitive status in older adults.

## Methods

### Participants

Data from 39 participants (female: n=27; age: mean 81.1, SD 5.9; range 69-90 years) with complete data were extracted from a larger, ongoing project [3]. All participants’ demographic and medical data were obtained through self-report, and no medical records or neuroimaging studies were available. For inclusion, participants were required to be English speakers and have no reported history of neurological conditions or severe psychiatric conditions. MCI status was determined by using criteria from past studies, namely, scoring ≥1 SD below the normative mean on 2 or more tasks within the same cognitive domain [8]. Following this criterion, 26% (10/39) of the participant sample were classified as having MCI; the remaining 29 participants were classified as cognitively intact. Table 1 presents summary statistics of the demographic and neuropsychological characteristics of the sample.

**Table 1.** Demographic characteristics and neuropsychological test performance of the study sample.

		Full sample (N=39)	Cognitively intact participants (n=29)	Participants with MCI^a^ (n=10)
**Demographic characteristics**				
	Age (years), mean (SD)	81.15 (5.95)	81.07 (5.84)	81.40 (6.59)
	Women, n (%)	27 (69)	18 (62)	9 (90)
	Men, n (%)	12 (31)	11 (38)	1 (10)
	Racial and ethnic minority participants^b^, n (%)	17 (44)	12 (41)	5 (50)
	Participants with depression, n (%)	3 (8)	3 (10)	0 (0)
**Neuropsychological test performance^c^, mean (SD)**				
	Mini-Mental State Exam (raw score)	28.85 (1.79)	29.17 (1.26)	27.90 (2.69)
	Digit Span Forward (T-score)	51.10 (9.79)	52.69 (9.25)	46.50 (10.34)
	Digit Span Backward (T-score)	52.51 (10.74)	54.55 (10.67)	46.60 (8.98)
	Trail Making Test A (T-score)	52.49 (8.69)	54.17 (6.11)	47.60 (12.92)
	Trail Making Test B (T-score)	51.72 (10.02)	52.96 (8.97)	48.50 (12.27)
	Frontal Assessment Battery (T-score)	47.36 (14.76)	51.21 (13.27)	36.20 (13.63)
	Controlled Oral Word Association Test (T-score)	56.97 (10.73)	58.59 (9.31)	52.30 (13.65)
	Animal Naming Test (T-score)	48.54 (11.27)	51.90 (7.83)	38.80 (14.28)
	Boston Naming Test–Short Form (T-score)	55.67 (11.00)	58.69 (8.16)	46.90 (13.72)
	Complex Figure Test–Copy (T-score)	41.67 (12.48)	43.55 (12.15)	36.20 (12.43)
	Complex Figure Test–Delayed Recall (T-score)	51.17 (18.76)	59.41 (12.81)	27.25 (10.94)
	HVLT^d^ (sum of trials 1-3; T-score)	52.18 (10.88)	55.79 (6.68)	41.70 (14.05)
	HVLT–Delayed Recall (T-score)	49.18 (13.23)	52.76 (9.71)	38.80 (16.89)
	HVLT Discrimination (T-score)	49.26 (12.03)	51.83 (9.61)	41.80 (15.53)

a MCI: mild cognitive impairment.

b The participants were African American, Asian, or Hispanic or Latino.

c With the exception of the Mini-Mental State Exam, of which the results are presented here as raw scores, all neuropsychological test scores were transformed to T-scores based on normative data.

d HVLT: Hopkins Verbal Learning Test.

### Ethical Considerations

This study was approved by the Kent State University Institutional Review Board (#20–300), and all procedures were completed in accordance with the ethical standards outlined in the Declaration of Helsinki. Upon entry into the study, all participants completed an informed consent process. Individuals demonstrating intact comprehension of study activities provided written consent and those with cognitive dysfunction provided assent and consent provided by a trusted other. Participants were assigned a randomly generated study identification number to protect confidentiality and privacy, and all materials were protected through multiple security measures. At the completion of the study assessment, participants were compensated with a gift card for their time.

### Neuropsychological Test Battery

To promote generalizability, participants completed a collection of commonly used neuropsychological tests of global functioning (Modified Mini-Mental State Exam [9]), attention (Digit Span Longest String Forward and Backward [10] and Trail Making Test A [11]), executive function (Trail Making Test B [11] and Frontal Assessment Battery), language (Controlled Oral Word Association Test [12], Animal Naming Test [12], and Boston Naming Test–Short Form [13]), visuospatial skills (Complex Figure Test–Copy [14,15]), and memory (Hopkins Verbal Learning Test–Revised [16] and Complex Figure Test–Delayed Recall [14,15]). Raw test scores were converted to T-scores using normative data to facilitate comparison to past work.

### Speech Tasks

Participants completed 3 picture description tasks and 2 expository tasks as part of the study protocol. Speech from these tasks was audio-recorded and then transcribed manually. Picture description tasks included the Cookie Theft task from the Boston Diagnostic Aphasia Exam [17], which depicts 2 children reaching into a cookie jar and a mother washing dishes. The other two pictures were drawn in a similar style, with one showing a man changing a lightbulb [18] and the other showing a kitten in a tree [19]. Expository tasks asked participants to describe an important person in their life (expository task 1) and a meaningful location or place (expository task 2). Importantly, the inclusion of a multiple categories of speech prompts (picture description tasks vs expository tasks) allowed us to examine whether different speech features can be reliably elicited across different types of tasks (eg, providing semantic structure in the form of a picture versus requiring memory retrieval and content generation).

A total of 16 lexical and semantic features were calculated based on the spontaneous speech generated from each task and were used as features in the analyses for word count, filler words, empty words, lexical frequency, the type-token ratio, the Honoré statistic, the Brunet index, speech rate, filler rate, definite articles, indefinite articles, pronouns, nouns, verbs, determiners, and content words. These features were chosen based on prior studies and clinical work that showed that these properties of speech production are often affected in persons with dementia or MCI [3]. All features were calculated automatically from transcripts of the participants’ speech, using Python (version 2.7.17) and the Natural Language Toolkit (version 3.2.1; Bird et al [20]). Table 2 shows the list of speech features and how they were defined; Table 3 shows the between-participant mean values for each linguistic feature that was computed from each speech sample.

**Table 2.** Operationalization of the speech features computed for each spontaneous speech task.

Speech feature	Operational definition
Word count	Total number of words spoken by the participant
Fillers	Number of filler words (eg, *um*, *uh*, and *hmm*) spoken by the participant; scaled by total word count
Empty words	Number of empty words (eg, *thing*, *place*, and *stuff*); scaled by total word count
Definite articles	Number of definite articles (*the*); scaled by total word count
Indefinite articles	Number of indefinite articles (*a* and *an*); scaled by total word count
Pronouns^a^	Number of pronouns; scaled by total word count
Nouns^a^	Number of nouns; scaled by total word count
Verbs^a^	Number of verbs; scaled by total word count
Determiners^a^	Number of determiners; scaled by total word count
Content words	Number of content words (defined as the words not in Natural Language Toolkit’s list of stop words); scaled by total word count
Frequency	Mean of the log of the frequency of all the words spoken by the participant
Type-token ratio	Ratio of unique words (types) to total words (tokens) spoken; used as a measure of lexical diversity
Honoré statistic	A measure of lexical richness based on the number of words that are produced exactly once
Brunet index	A measure of lexical diversity and richness that is less biased by the length of the text
Speech rate	Speech rate was computed as words per second, counting all words, nonwords, and partial words the speaker produced divided by the total elapsed time of the speech
Filler rate	Filler rate was computed as words per second, counting all filler words (as defined above) divided by the total elapsed time of the speech

a Computed using the Penn Treebank part of speech tags within the Python Natural Language Toolkit module (Bird et al [20]).

**Table 3.** Mean values for the computed speech features across the five speech tasks for the full sample.

Speech feature	Value, mean (SD)				
	Expository task 1 (person)	Expository task 2 (place)	Picture description task 1 (cookie theft)	Picture description task 2 (lightbulb)	Picture description task 3 (cat in tree)
Word count	632.18 (316.32)	531.64 (412.87)	290.82 (172.77)	233.92 (117.47)	222.69 (106.25)
Number of fillers	1.23 (0.61)	0.98 (0.57)	0.63 (0.47)	0.54 (0.36)	0.43 (0.33)
Number of empty words	0.20 (0.16)	0.51 (0.36)	0.18 (0.12)	0.28 (0.18)	0.15 (0.14)
Number of definite articles	0.60 (0.36)	1.00 (0.51)	1.38 (0.45)	0.94 (0.3)	1.35 (0.33)
Number of indefinite articles	0.79 (0.29)	0.69 (0.33)	0.86 (0.3)	1.19 (0.36)	0.84 (0.28)
Number of pronouns	3.29 (0.95)	2.19 (1.1)	1.18 (0.6)	1.15 (0.54)	0.94 (0.54)
Number of nouns	5.26 (1.49)	4.80 (1.66)	4.14 (1.2)	3.69 (0.92)	3.38 (0.79)
Number of verbs	5.15 (1.47)	4.18 (1.57)	3.42 (1.07)	3.22 (0.91)	3.03 (0.87)
Number of determiners	1.84 (0.69)	2.15 (0.87)	2.54 (0.7)	2.46 (0.56)	2.44 (0.43)
Number of content words	11.89 (3.04)	10.31 (3.53)	8.28 (2.55)	7.44 (1.97)	7.04 (1.84)
Frequency^a^	5.68 (0.41)	5.80 (0.49)	5.32 (0.43)	5.54 (0.46)	5.76 (0.55)
Type-token ratio	0.41 (0.08)	0.43 (0.09)	0.48 (0.09)	0.50 (0.08)	0.48 (0.05)
Honoré statistic	5.16 (3.15)	6.29 (3.78)	7.85 (2.42)	8.40 (2.49)	9.48 (3.16)
Brunet index	13.14 (1.13)	12.98 (1.4)	12.23 (1.22)	11.92 (1.15)	12.11 (0.79)
Speech rate^b^	2.20 (0.37)	2.35 (0.37)	2.31 (0.35)	2.31 (0.33)	2.53 (0.39)
Filler rate^c^	0.11 (0.05)	0.10 (0.06)	0.09 (0.06)	0.08 (0.05)	0.07 (0.06)

a Mean of the log of the frequency of all the words spoken by the participant.

b Words per second, counting all words, nonwords, and partial words the speaker produced divided by the total elapsed time of the speech.

c Words per second, counting all filler words divided by the total elapsed time of the speech.

### Procedures

Participants completed all neuropsychological tests and speech elicitation tasks during a single study visit that lasted approximately 75 minutes. After providing written informed consent, participants were administered the neuropsychological test battery in a fixed order, under the supervision of a licensed clinical neuropsychologist. The aforementioned spontaneous speech tasks were then completed. The session concluded after participants were provided with a debriefing statement and compensated for their time.

### Data Analyses

#### Overview

As several of the speech features were measured on different scales (eg, lexical frequency was computed as number of words per million, parts of speech features were scaled by the total word count, the total number of words was a raw count, etc), the raw values for each speech feature were converted to *z*-scores to enable interfeature comparisons. The *z*-scoring of each participant’s speech feature values was performed separately for each speech feature, by task (eg, picture description task 1, picture description task 2, expository task 1, etc) and cognitive status group (ie, MCI vs cognitively intact). The *z*-scored values for each speech feature were then used in the following analyses.

#### Intraindividual Variability Across Instances of the Same Speech Task

To assess the degree to which a given speech feature remained consistent for each participant across multiple instances of the same speech elicitation task, pairwise Pearson *r* correlations were computed between each feature and itself within each task type. Afterward, to examine the influence of cognitive dysfunction on these indices, correlations were computed separately for participants with MCI and cognitively intact participants. For example, a paired correlation was computed, for all participants in the MCI group, between the *z*-scored word count values for expository task 1 and the *z*-scored word count values for expository task 2. For the picture description tasks, the correlations were averaged over the three pairwise correlations of picture description tasks (task 1–task 2, task 1–task 3, and task 2–task 3). All averaging of correlation values was performed after the Fisher *z* transformation of the Pearson *r* correlation coefficients [21]. After averaging was completed, Fisher *z* values were back-transformed to Pearson *r* values for reporting.

In order to determine whether these mean correlations were significantly larger than what would be expected for any two given measurements of the same linguistic feature, we used resampling methods. Null distributions of correlations were created for each task type by randomly pairing each participant’s speech feature values with values for the same speech features from a different, randomly selected participant within the same group (MCI or cognitively intact group). These correlations show how much a participant’s value for one feature correlates with a different person’s value for the same feature and thus can be used as a baseline for the expected size of within-feature correlations, if there is no additional effect from within-participant reliability. This resampling procedure was repeated 10,000 times for each of the four null distributions, which were then used as the distribution against which the true correlation values were compared to compute their *P* value.

#### Intraindividual Variability Across Multiple Speech Tasks

Intraindividual variability was calculated for each speech feature by computing the SD of a participant’s *z*-scores for a given speech feature across all 5 tasks (eg, the SD of a participant’s *z*-transformed word count values across expository task 1, expository task 2, picture description task 1, picture description task 2, and picture description task 3). Weighted averages of the variance of these SDs were then computed as an index of intraindividual variability. These SD values were then averaged over participants for each of the 16 speech features, as shown in the following formula (larger values reflected greater intraindividual variability):


$$\sqrt{\frac{SD_{ET1}^{2}\;+\;SD_{ET2}^{2}\;\;+SD_{PDT1}^{2}+\;SD_{PDT2}^{2}+\;SD_{PDT3}^{2}}{N}}$$

## Results

### Intraindividual Variability Across Instances of the Same Speech Task

In the picture description tasks, the mean within-participant correlation between the 16 speech features and themselves across the three possible pairwise comparisons (task 1–task 2, task 1–task 3, and task 2–task 3) was high (MCI group *r*: mean 0.6555, SD 0.2867; cognitively intact group *r*: mean 0.6440, SD 0.2997). The strength of the correlation was not statistically different between the two cognitive status groups (*t*_30_=0.4351; *P*=.66; 95% CI −0.17 to 0.26).

In the expository tasks, the mean within-participant correlation between the speech features and themselves was similarly high for the MCI group (*r*: mean 0.6101, SD 0.3679) but lower for the cognitively intact group (*r*: mean 0.4971, SD 0.3586), although this between-group difference did not reach statistical significance (*t*_30_=1.363; *P*=.18; 95% CI −0.09 to 0.45).

We then examined whether these correlations were significantly different from what might be expected between any two given linguistic measures, using the resampling procedure described in the *Methods* section. The average correlation for each of the null distributions was extremely close to 0 (MCI group picture description task: *r*=0.0022; cognitively intact group picture description task: *r*=−0.0002; MCI group expository task: *r*=0.0004; cognitively intact group expository task: *r*=0.0002), and all 4 true within-participant correlations were significantly larger than what was expected by chance based on these null distributions (all *P* values were <.001).

Notably, mean correlations varied substantially across different speech features (Table 4). Some speech features showed consistently strong correlations, suggesting high reliability (such as speech rate, Brunet index, and number and rate of filler words), while others showed lower reliability (such as empty words, definite and indefinite articles, determiners, and pronouns).

**Table 4.** Reliability values for the speech features.

			Total words	Fillers	Empty words	Definite articles	Indefinite articles	Pronouns	Nouns	Verbs	Determiners	Content words	Frequency	Type-token ratio	Honoré statistic	Brunet index	Speech rate	Filler rate
**Reliability analysis of each task type^a^**																		
	**Expository tasks**																	
		Full sample	0.581	0.75	0.372	0.188	0.499	0.566	0.580	0.614	0.368	0.667	0.480	0.720	0.325	0.748	0.895	0.769
		MCI^b^ group^c^	0.728	0.782	0.676	0.285	0.321	0.607	0.740	0.705	0.313	0.807	0.601	0.814	–0.04	0.817	0.884	0.721
		Cognitively intact group^d^	0.382	0.714	–0.039	0.087	0.643	0.521	0.357	0.503	0.421	0.455	0.337	0.587	0.613	0.659	0.905	0.809
	**Picture description tasks**																	
		Full sample	0.814	0.756	0.422	0.461	0.545	0.674	0.722	0.746	0.647	0.870	0.774	0.721	0.245	0.784	0.79	0.73
		MCI group	0.823	0.746	0.557	0.513	0.626	0.521	0.689	0.753	0.661	0.907	0.835	0.691	0.271	0.763	0.798	0.72
		Cognitively intact group	0.805	0.765	0.265	0.406	0.452	0.785	0.752	0.739	0.631	0.821	0.696	0.749	0.218	0.803	0.782	0.74
**Reliability analysis of all tasks^e^**																		
	Full sample		0.712	0.616	0.881	0.848	0.817	0.727	0.754	0.734	0.762	0.71	0.809	0.685	0.723	0.755	0.721	0.488
	MCI group		0.647	0.641	0.754	0.777	0.775	0.806	0.693	0.65	0.696	0.61	0.679	0.63	0.596	0.593	0.573	0.497
	Cognitively intact group		0.733	0.607	0.921	0.871	0.831	0.698	0.774	0.761	0.783	0.741	0.850	0.702	0.762	0.804	0.765	0.485

a This section reports the mean within-participant correlations between each speech feature and itself for each task type and group. All averaged correlations were converted to Fisher *z* values before averaging and back-transformed to Pearson *r* values for reporting.

b MCI: mild cognitive impairment.

c The MCI group includes persons diagnosed with MCI.

d The cognitively intact group includes persons diagnosed as not having MCI.

e This section reports the SDs of *z*-scored values for each speech feature computed over all 5 tasks, which were averaged across participants within each group. Larger values reflect more intraindividual variability.

### Intraindividual Variability Across Multiple Speech Tasks

The amount of variability in each speech feature for each participant additionally varied as a function of speech feature and group (Table 4). The lowest amount of intraindividual variability was exhibited by speech rate and filler rate for the cognitively intact group and by speech rate for the MCI group. The largest amount of intraindividual variability differed somewhat between the MCI and cognitively intact groups; for example, definite and indefinite articles showed high between-participant variability for both groups, whereas empty words showed numerically higher variability for the cognitively intact group and pronouns showed numerically higher variability for the MCI group.

## Discussion

Some evidence suggests that there is greater variability in performance on traditional cognitive screening measures (eg, Mini-Mental State Exam, Clock Drawing Test, etc) among persons with MCI [22]. Although such variability itself can be a useful marker of MCI [23], variability can also make results harder to replicate and lower statistical power. Given that spontaneous speech (1) is affected in MCI and (2) may be useful for distinguishing healthy controls from individuals with MCI and ADRD [3,4,24,25], it was therefore important to establish the degree of variability (or stability) of spontaneous speech in individuals with and without MCI. The results from this preliminary study demonstrate that spontaneous speech is generally consistent in both individuals with MCI and cognitively intact older adults, as individuals maintained their lexical-semantic characteristics of speech across multiple tasks. Such findings provide initial evidence that properties of an individual’s spontaneous speech are sufficiently “reliable” to be viewed as trait-like features and encourage continued investigation into the validity of speech analysis data as digital biomarkers of cognitive status.

Given the importance of the early detection of cognitive decline, future studies may be enhanced by examining the potential value in using a combination of indices from spontaneous speech to predict cognitive status—not just lexical-semantic features. For example, acoustic-phonetic aspects of speech, such as prosodic measures, pause duration, or loudness, are also impacted by ADRD and can distinguish healthy groups from clinical groups [26,27]. Changes in the syntax and coherence of speech are found in persons with advanced ADRD and can be reliably detected [28,29]. There is also evidence that subtle changes in extrapyramidal function predict incipient MCI and Alzheimer disease [30], and recent technological advances can automatically quantify these changes in short video clips of an individual, suggesting the possibility of extending this work into measuring behavior in video calls or videoconferencing (eg, FaceTime and Zoom) or via mobile apps [31]. It is possible that a combination of multiple speech features and video analysis may prove more sensitive to early cognitive decline than a single category of linguistic features; thus, further work in this area is needed. More research should also be directed at determining the reliability of such features in other neurological brain disorders for which some aspects of language have been shown to be associated with decline, such as Parkinson disease [32].

Despite encouraging findings, this study is limited in several important ways. The sample size was modest, the analysis was cross-sectional in nature, and we only assessed speech and cognitive function during a single testing session. Although several findings were statistically significant despite the modest sample size, the nonsignificant group difference in intraindividual variability across instances of the same speech task type (expository tasks; *P*=.18) may have been underpowered due to the small sample. Therefore, future research on the consistency of speech tasks for assessing MCI should ensure sufficient power. Furthermore, prospective studies with larger and more diverse samples are needed to clarify the feasibility of using automated speech analysis (Soroski et al [33] used such analyses in research settings and for at-home monitoring of cognitive function), though several studies on automatic speech analysis have shown such analyses to be promising [5,34,35]. Such findings will provide key insight into the stability of spontaneous speech over longer intervals (eg, weeks to months). It is also possible that the prospective monitoring of speech changes may help to overcome some of the limitations (ie, higher rates of misclassification of cognitive status) found in existing cognitive screening instruments for diverse populations [36,37] and facilitate early identification. This study is also limited in that effects of depression were not able to be explored. Future studies should examine the possible contributions of depression and anxiety to spontaneous speech in older adults, given that mental health conditions are common in older adults [38] and that depression may also alter speech content [39] and vocal features [40]. Finally, an important limitation of this study is that participants’ cognitive status (MCI and cognitively intact), as well as other potentially relevant medical conditions (eg, depression), was based on a self-report of their history of diagnosed neurological conditions. Detailed information regarding specific etiology was not available or objectively assessed, limiting the strength of our conclusions (including the possibility that MCI was not due to Alzheimer disease). Future studies on the reliability of speech as a marker of MCI should incorporate more comprehensive neurological evaluations to ensure that the assessment of speech reliability is valid (eg, neuroimaging and other biomarkers).

In summary, our findings suggest that lexical-semantic aspects of spontaneous speech are similarly reliable in older adults with and without MCI. This finding is an essential first step toward the widespread use of speech biomarkers as a low-burden method for cognitive monitoring and the facilitation of the early detection of neurodegeneration in persons at risk for ADRD.

## Abbreviations

ADRD: Alzheimer disease and related dementias
MCI: mild cognitive impairment
